# A New Potentiometric Sensor for Determination and Screening Phenylalanine in Blood Serum Based on Molecularly Imprinted Polymer

**Published:** 2019

**Authors:** Zahra Bangaleh, Hayedeh Bagheri Sadeghi, Soltan Ahmad Ebrahimi, Parvaneh Najafizadeh

**Affiliations:** a *Department of Pharmacology, School of Medicine, Iran University of Medical Science, Tehran, Iran.*; b *Department of Chemistry, Faculty of Science, Tehran Central Branch, Azad University, Tehran, Iran.*

**Keywords:** Phenylalanine, Potentiometric sensor, Molecular electrochemistry, MIPs

## Abstract

Methods routinely utilized for detection of phenylalanine in new-born blood consist of enzymatic assays, lacking sensitivity and HPLC assays which are expensive and time-consuming to conduct. We, here, report for the first time, the construction of a phenylalanine sensitive electrode, on the basis of a selective molecularly imprinted polymer, offering sensitivity, economy and ease of use for the measurement of phenylalanine .The sensor was constructed of a graphite-rod electrode which was coated by MIP embedded polymer base made from polyvinyl chloride and plasticizer mixture, dissolved in THF. At optimized conditions the electrode revealed a Nernstian response 29.73 ± 1.0 mV decade^-1^ in a concentration range of 1 × 10⁻⁸ to 1 × 10^-4^ M with detection limit of 5 × 10⁻⁹ M. The potential response of the electrode was constant in the pH range of 4.0–7.5. The electrode unfolded a response time of ~20 sec. The selectivity coefficient of the sensor towards a number of different amino acids with molecular similarities and some metal ions was evaluated. The sensor was successfully used for determination of phenylalanine in blood serum and the results were in good compatibility with HPLC method.

## Introduction

**Figure 1:**
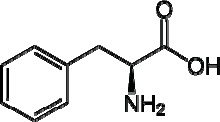
L-Phenylalanine (LPA)

L-Phenylalanine is an α-amino-acid with the formula C6H5CH2CH(NH2)COOH, molar mass of 165.19 g/mol and pK a 9.13 (1) (Figure 1). This essential amino-acid is classified as nonpolar because of the hydrophobic nature of the benzyl side chain. L-Phenylalanine (LPA) is an electrically neutral amino-acid used to biochemically form proteins. LPA is found naturally in the breast milk of mammals. It is used in the manufacture of food and drink products and sold as a nutritional supplement for its reputed analgesic and antidepressant effects. It is a direct precursor to the neuromodulator phenethylamine, a commonly used dietary supplement. Phenylketonurea is an inborn metabolic disorder which is characterized by increased serum phenylalanine. This hyperphenylalaninemia is mainly due to the abnormally low activity of phenylalanine hydroxylase, the enzyme responsible for the conversion of phenylalanine to tyrosine. As a result of this genetic defect, phenylalanine and its metabolites from alternative biochemical pathways, accumulate in the brain leading to tissue damage which manifests itself as abnormal neurological development and mental retardation. There is no cure for this abnormality and the most effective treatment for the affected individuals is early detection followed by strictly limiting phenylalanine intake in food. As a result, in many countries hyperphenylalaninemia screening programs for the newborn have been run by health agency for many years. The frequency of this abnormality in the population varies greatly from one country to another, being low in Finland (1:200000) and Japan (1:120000) while it is much higher in Turkey (1:2600) and Iran (1:3672) (2).

**Table 1:** Optimization of membrane ingredient

**Membrane No.**	**MIP**	**PVC**	**O-NPOE**	**DOP**	**NB**	**NaTPB**	**KTpCIPB**	**OA**	**THF**	**Linear Range**	**Slope (mV/decade)**
1	6.5	31	-	-	61	1.5	-	-	1.5	10-6 – 10-7	2.0
2	6.5	31	61	-	-	1.5	-	-	1.5	10-4 – 10-7	11.3
3	8.5	30	60	-	-	1.5	-	-	1.5	10-4 – 10-7	16.6
4	8.5	30	-	60	-	1.5	-	-	1.5	10-4 – 10-7	12.3
5	10	30	-	-	59	1	-	-	1.5	10-4 – 10-8	4.0
6	10	30	-	-	59	-	1	-	1.5	5 × 10-4 – 5 × 10-7	9.3
7	10	30	-	-	59	-	-	1	1.5	10-5 – 10-8	2.2
8	10	30	-	59	-	1	-	-	1.5	10-4 – 10-8	12.9
9	10	30	-	59	-	-	1	-	1.5	10-4 – 10-8	5.3
10	10	30	59	-	-	-	-	1	1.5	10-5 – 10-8	16.5
11 [a]	10	30	59	-	-	1	-	-	1.5	10-4 – 10-8	29.7
12 [b]	10	30	59	-	-	1	-	-	1.5	10-4 – 10-8	3.6
13 [c]	-	30	59	-	-	1	-	-	1.5	10-3 – 10-8	_

[a] MIP. [b] NIP. [c] Blank.

**Table 2:** Selectivity coefficient values using matched potential method

**Interference**	log K_AB_ pot
Tyrosine	1.65 × 10-3
Tryptophan	4.95 × 10-3
Histidine	6.18 × 10-3
Valine	3.8 × 10-3
Aspartic Acid	8.25 × 10-3
Leucine	3.09 × 10-3
Isoleucine	8.25 × 10-3
Glycine	4.5 × 10-3
Ca2+	3.53 × 10-3
Na+	4.95 × 10-3
Mg2+	1.65 × 10-3
K+	4.95 × 10-3

**Table 3:** Recoveries obtained by potentiometric method in respect to standard HPLC method

**ID**	**Potentiometric response (µM)**	**HPLC response (µM)**	**Recovery (%)**
1	1332	1291	103
2	646	624	103
3	580	563	103
4	558	557	100
5	460	455	101
6	362	352	103
7	162	163	99
8	57	60	95
9	142	151	93
10	44	48	91
11	53	67	79
12	190	194	98
13	394	418	94
14	74	91	82
15	603	642	94
16	222	212	104
17	168	170	99
18	175	200	87
19	61	79	78
20	1220	1091	111
21	1551	1376	112

**Figure 2:**
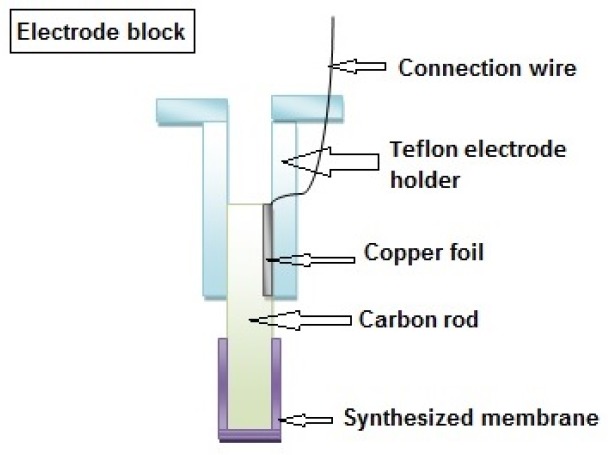
Block diagram of proposed electrode

**Figure 3:**
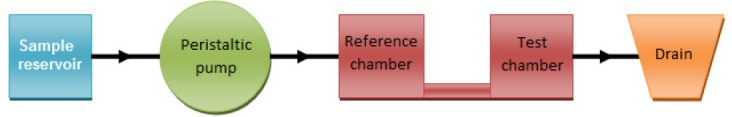
Schematic diagram of the designed system

**Figure 4:**
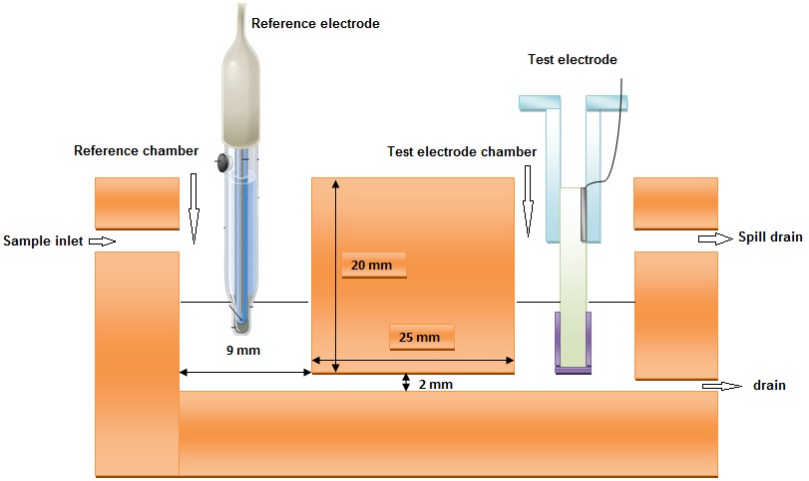
Schematic diagram of the cell

**Figure 5:**
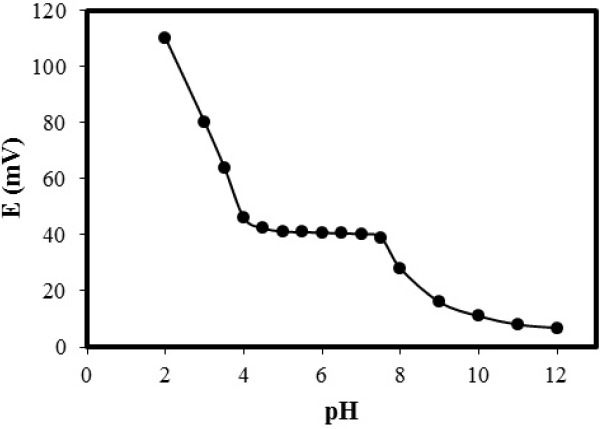
Effect of pH on the response of optimized electrode

**Figure 6:**
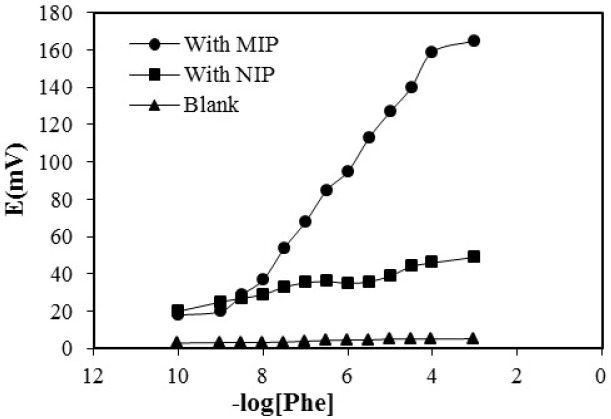
Calibration curve for proposed electrode with optimized composition based on MIP NIP, and blank (with no polymer)

**Figure 7:**
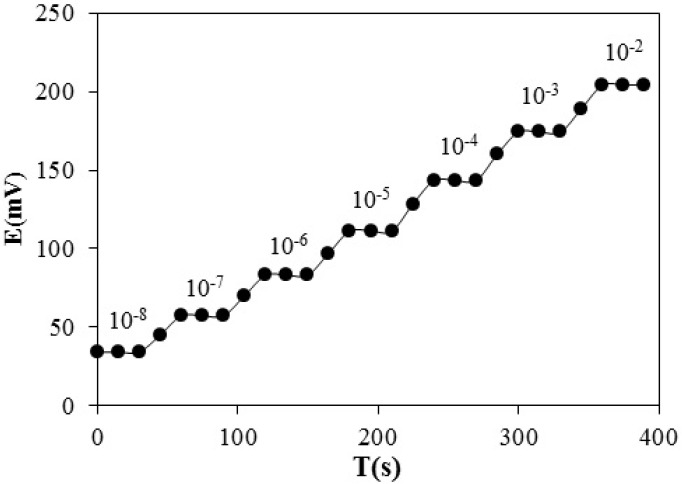
Dynamic response of the electrode for step changes in phenylalanine concentration

**Figure 8:**
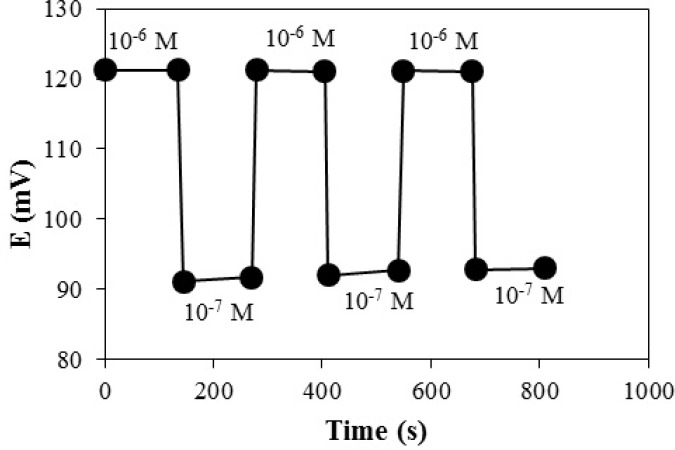
Response characteristics of proposed electrode for high to low sample cycle

**Figure 9:**
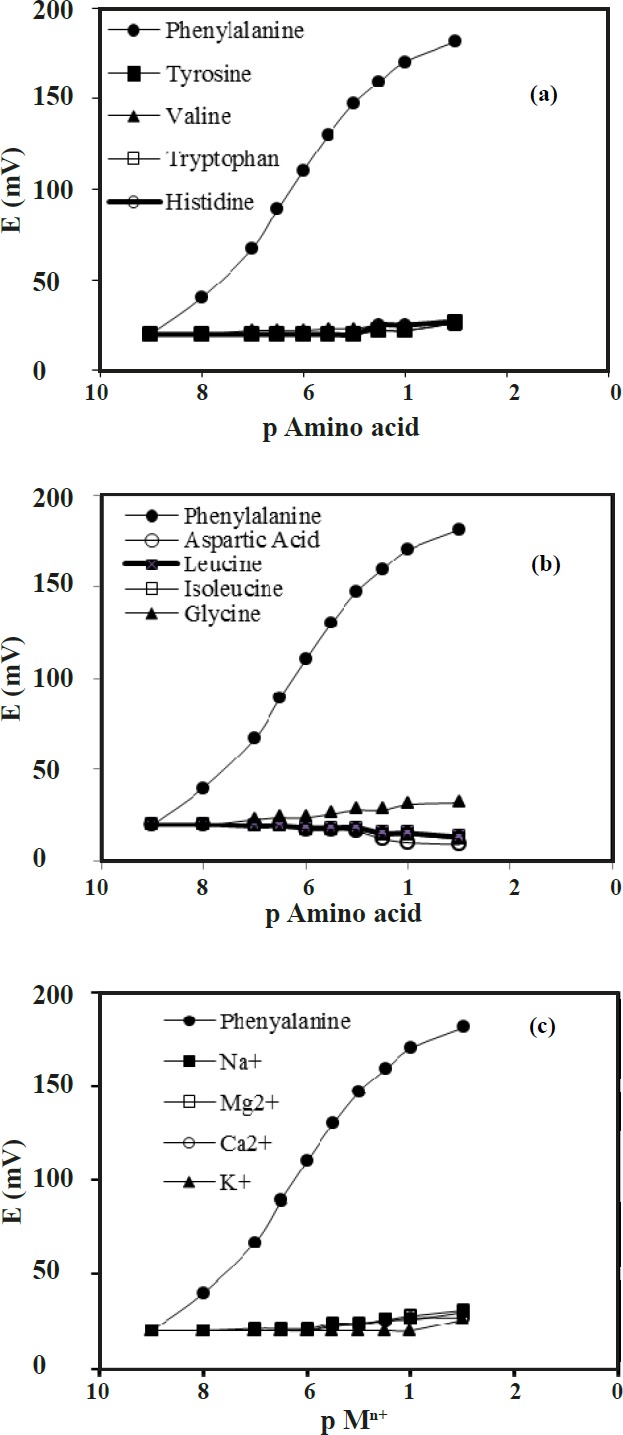
(a, b and c) Potential response of proposed electrode towards a number of different amino acids with molecular similarities and some metal ions

**Figure 10:**
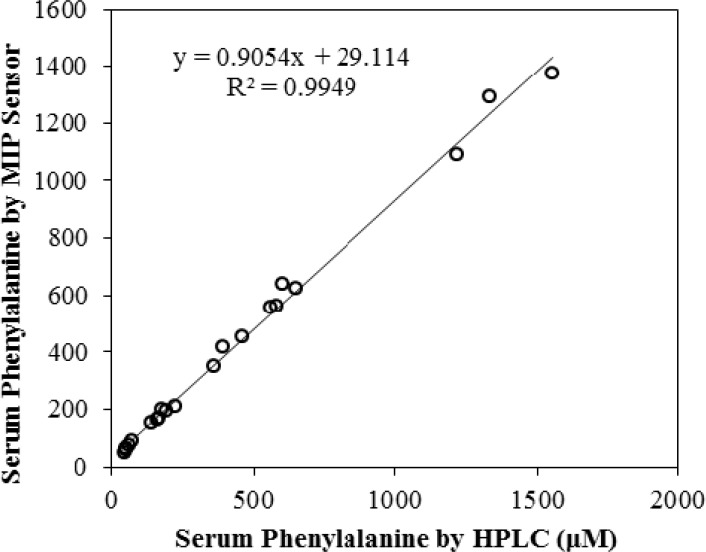
Correlation of the potentiometric and HPLC measurements of phenylalanine in 21 blood serum samples

There are few methods for determination of phenylalanine, such as Gutrie test, flourometric and HPLC-Tandem Mass Spectrometry (2-5) which is the method of choice as it offers great specificity and excellent sensitivity for the detection of phenylalanine in serum or other matrices. As mentioned above, there is no cure for this disease and early detection is of paramount importance for effective suppression of symptoms later in life. In many countries access to modern mass spectrometric or chromatographic methods is not immediately available to all health centers and therefore, detection of phenylketonuria (PKU) requires the sample to be sent to central labs. This process is expensive and time consuming often taking days for the sample to reach the lab and results to get back. Thus the need is strongly felt for a method which is simple enough to carry out in all health centers/maternity units and also has suitable analytical characteristics with regards to sensitivity, selectivity and reproducibility. Electrochemical sensors appear to be good candidates.

Molecularly imprinted polymers (MIPs) are attractive as versatile and inexpensive materials capable of molecular recognition. MIPs consist of highly cross-linked polymers that are synthesized in the presence of a template molecule. Subsequent removal of the template leaves a cavity that retains affinity and selectivity for the template.

The cavity is analogous to the ‘active site’ of enzymes. These MIPs have shown to be useful in enantioseparations, catalysis, solid-phase extraction, drug delivery chromatography and in the preparation of sensors specific for the analytes of interest, using a variety of sensing methods (6). Over the two decades, imprinted polymers have attracted a broad interest from the scientists engaged in sensor development. This attention can be attributed to the serious potential advantages of the MIPs usage in place of natural receptors and enzymes, such as their superior stability, low cost, high selectivity, and easy preparation. A few examples of detection methods using imprinted polymers include: fluorescence (7), luminescence (8), surface Plasmon resonance (9-10) quartz crystal microbalance (10), impedance measurement (11), induced scintillation (12), field effect transistor (13), optical approaches (14-15), and acoustic wave sensors (16). Currently, there is an increasing number of the MIP reports on electrochemical sensors with capacitive (17-18), conductometric (19), amperometric (20-21), and voltammetric (22-24) transduction. Despite the relatively simple transduction of the potentiometric signal, only limited reports on design of the potentiometric sensors based on the molecular imprinted technology can be found in literature (25-30).

In this work we have developed a new ion selective electrode based on molecularly imprinted polymers for determination of phenylalanine, which to our knowledge is the first reported electrode of this kind.

## Experimental

*Materials and Instruments*

L-phenylalanine (LPA), tyrosine, valine, tryptophane, histidine, aspartic acid, leusine, iso-leusine, glycine, polyvinyl chloride (PVC), dibutyl phetalate (DBP), dioctylphetalate (DOP), nitrobenzene (NB), ortho-nitrophenoloctyl ether (O-NPOE), sodium tetraphenyl borate (NaTPB), oleic acid (OA), potassium tetrakis (KT_p_ClPB), and ethylene glycol dimethacrylate (EDMA) were obtained from Sigma-Aldrich (Milwaukee, USA).

2,2’-Azo-bis-iso-butyronitrile (AIBN)was obtained from Acros (Geel, Belgium). Tetrahydrofuran (THF), acetonitrile (ACN), methanol (MeoH) and acetic acid were all of HPLC grade and purchased from Aldrich (USA), and used without any further purification. The potential measurements were performed by means of Metrohm-629 pH/mv meter.

*Preparation of Phenylalanine Imprinted Polymer*

The molecularly imprinted polymer against L-phenylalanine was synthesized as reported previously (31). A mixture of acetonitrile (3 mL) and glacial acetic acid (0.6 mL) containing 1 mmol L-Phenylalanine and 1.2 mM trichloroacetic acid (TCA) was prepared. After sonication for 10 min, 2 mL of acetonitrile containing 0.5mmol 2,2’-Azo-bis-iso-butyronitrile (AIBN) and 20 mmol ethylene glycol dimethacrylate (EDMA) was added. The solution was mixed for 5 min in a thick walled glass tube and sparged with argon for a further 5 min. The reaction vessel was sealed and the polymerization reaction was allowed to proceed for 24 h at room temperature. The polymer mass obtained was ground into a powder and washed with MeOH (four times) and 10% w/v TCA (once). The MIP was again washed by resuspension in 5% acetic acid solution and centrifugation. This step was repeated (usually about 25 times) until no L-phenylalanine could be detected by HPLC. The polymer was washed with distilled water, by centrifugation and resuspension, until the pH of the supernatant equaled that of water. After drying, grinding, and passing through a series of sieves (particle size less than 74 µm), the polymer was used to prepare PVC film electrodes. The NIP was synthesized in the absence of phenylalanine with the same procedure as MIP.

*Electrode Preparation*

The sensor was prepared by thoroughly mixing 10 mg of MIP or NIP, 30 mg of PVC, 59 mg of O-NPOE as plasticizer and 1 mg NaTPB as additive in 2 mL of THF. The resulting mixture was completely dissolved and left for about 20 min until the extra solvent evaporated and a concentrated mixture was obtained. A carbon rod was then dipped into the mixture until it was covered with a thin layer of the mixture. Then, the modified carbon rod was left for few hours to dry and finally a piece of copper foil and a wire was attached to the upper end of the electrode to make the electrical connection possible Figure 2.

Since the aim of the research was to measure small amounts of blood serum, it was decided to make a special cell which can hold only few milliliters (~ 2 mL each compartment) of the sample. This cell is constructed of two small compartments connected to each other by a narrow channel and has a sample inlet and drain outlet. The cell dimensions are shown in Figure 3.The sample is pumped in by a peristaltic pump Figure 4. It must be mentioned that the pump is only used to transfer the sample into the cell compartments and measurements are performed in quiescent solution.

*Emf Measurements*

The performance of the sensor was investigated by measuring the emf values of various phenylalanine standard solutions. Potentiometric evaluation of the electrodes was carried out using the following cell:

Ref. electrode | |test solution | MIP membrane coated carbon rod electrode 

Activity coefficients of ions in aqueous solutions were calculated according to the Debye-Hückel equation.

## Results and Discussion

*Optimization of the Membrane*

Effect of different amounts of PVC, MIP, plasticizer, and additive were investigated on performance of membrane. Different types of plasticizers such as O-NPOE, DOP, DBP, and NB were used and to reduce the ohmic resistance, additives KTpCIPB and NaTPB were examined. The results are listed in Table 1. The best response was exhibited by the membrane no. 7 incorporating MIP, PVC, O-NPOE, NaTPB, and THF as solvent in the ratio of 10:30:59:1(MIP: PVC: O-NPOE: NaTPB in %wt) respectively. 

*Effect of pH*

The sample pH effect on potential response of the sensor was tested with a 10^-6 ^M standard solution of LPA. 

The pH of solutions was adjusted between 2-12 by addition of concentrated NaOH or HCl solution and potentials were recorded. Results are shown in Figure 5. A constant potential response in pH range of 4-7.5 can be observed. At alkaline pH values formation of hydroxyl amino-acid complex and in acidic solution (pH less than 4.0) protonation of phenylalanine are most probably responsible for this behavior. Therefore, it was decided to make all potential measurements in pH 6.0.

*Calibration Curve*

The potential response curves of MIP-, NIP and the blank membrane are shown in Figure 6. 

The MIP membrane shows a Nernstian response of 29.73 ± 0.92 mv per decade over the concentration range of 1 × 10⁻⁸ to 1 × 10⁻^⁴^ M with a detection limit of 5 × 10^-9^. At very high concentration of phenylalanine, calibration curve levels off due to saturation of imprinted phenylalanine. This is not considered as a disadvantage since, determination of very low concentration of phenylalanine is needed for evaluation of hyperphenylalaninemia in new-born blood.

*Response Time and Reversibility of the Electrode Response*

 Response time of an ion-selective electrode is defined as the average time required for the sensor to reach ± 1 mv of the magnitude of the equilibrated potential signal after successively immersing the electrode in a series of ion solution, each having a 10 fold concentration difference. The dynamic potential response with time is presented in Figure 7. As seen, the electrode reaches to its equilibrium response in a short time (<20 sec) in all concentrations. To evaluate the reversibility of the electrode, a similar procedure with opposite direction was adopted. 

The measurements were performed in the sequence of high-to-low sample concentrations, and the results are shown in Figure 8. It shows that the potentiometric responses of the sensor was reversible and had no memory effect, although the time needed to reach equilibrium values were longer than that of low-to-high sample concentration.

*Interference Studies*

The existence of the interfering ions affects the response behavior of ion-selective electrodes. For this reason, the term of selectivity coefficients, K_Sel_, is employed to describe this phenomenon. In our investigation, the calculation of the selectivity coefficients was conducted with the help of the matched potential method (MPM). In matched potential method, the potentiometric selectivity coefficient is defined as the activity ratio of primary and interfering ions that give the same potential change under identical conditions. At first, a known activity (a ^‘^_A_) of the primary ion solution is added into a reference solution that contains a fixed activity (a_ A_) of primary ions, and the corresponding potential change (ΔE) is recorded. Next, a solution of an interfering ion (B) is added to the reference solution until the same potential change, (ΔE) is recorded. The change in potential produced at the constant background of the primary ion must be the same in both cases. The selectivity coefficient of the sensor towards a number of different amino acids with molecular similarities and some metal ions was evaluated. The results are listed in Table 2, and shown in Figure 9.

*Analytical Application*

The proposed sensor was directly used as an indicator electrode for determination of phenylalanine in human blood serum. Human blood was collected from 21 thoroughly controlled blood donors (new-born up to 25 years old) from a diagnostic laboratory in Tehran. The red blood cells were separated by centrifugation and then the blood serum was frozen at -20 °C. Before use; the blood serum was thawed for about an hour at room temperature. Sample solutions for direct potentiometric determination were prepared by dilution of 10 µL of blood serum to 5 mL by deionized water. The potential was measured and the concentration of phenylalanine was calculated from calibration curve line-equation. Each experiment was repeated three times (n = 3) and the mean values are listed in Table 3. Dilution coefficient was taken into account in concentration calculation of each sample. To verify the results and obtain the accuracy of the potentiometric method, the measurements were made by standard HPLC method used in medical diagnostic laboratories.

*HPLC assay of phenylalanine*

The HPLC analysis of serum phenylalanine was carried out using an assay based on the method described by Hilton (32). Briefly, 50 µL of 500 mM methyl-DL-phenylalanine, as internal standard, was added to 50 µL serum sample. The solution was mixed for 30 sec. One-hundred-fifty µL 5% perchloric acid was added to the solution and the mixture was vortex mixed for 30 sec. Thirty µL of 2 M KOH was added and mixed for 10 sec. The cloudy solution was allowed to stand at 4 °C for 10 min. The solution was centrifuged at 4000×g and 40 µL of the supernatant was injected onto the HPLC column. The chromatography conditions were as follows: Isocratic separation was affected using a mixture of Acetonitrile: 2 mM sodium dihydrogen phosphate buffer (pH 3.5) (3:97 by volume), pumped at a flow rate of 1 mL per min through a 4.6 × 150 mm, 5 µm, ODS-B, and Tracer Excel column (Teknokroma, Barcelona, Spain). The column eluate was monitored at 214 nm. The chromatograph consisted of a Young Lin 9100 HPLC system (Young Lin, Korea). Chromatograms were recorded using Autochro-3000 software package (Young Lin, Korea). The HPLC assay was calibrated using phenylalanine-spiked serum samples over the range of 10 to 1250 µM. Assay validity was regularly checked using commercial control sera. The recovery results are summarized in Table 3 and correlation coefficient between the two methods (R^2 ^= 0.9949) is shown in Figure 10 which both indicates the excellent performance of the sensor.

## Conclusion

In this work we have developed a new ion selective electrode based on molecularly imprinted polymers for determination of phenylalanine in blood serum, which to our knowledge is the first reported electrode of this kind. The sensor was constructed of a carbon rode which was coated by a membrane consisting of phenylalanine MIP, o-NPOE as plasticizer and NaTPB as additive. A special cell was designed, constructed and used instead of usual beakers, which permitted small volumes of sample intake. At optimized conditions the electrode exhibited a nernstian response 29.73 ± 1.0 mV decade⁻¹ in a concentration range of 1 × 10⁻⁸ to 1 × 10⁻^⁴^ M with detection limit of 5 × 10⁻⁹ M. The potential response of the electrode was constant in the pH range of 4.0–7.5. The electrode unfolded a response time of ~20 sec. The selectivity coefficient of the sensor towards a number of different amino acids with molecular similarities and some metal ions was evaluated. The sensor was successfully used for determination of phenylalanine in blood serum and the results were in good compatibility with HPLC method and offered the sensitivity required for the detection of phenylalanine in biological fluids. The proposed sensor is easy to use, fast, and cost-effective.
